# A Multi‐Sector Mixed Methods Study of Stroke Services in the Philippines: Insights From Government Officials and Organisational Leaders

**DOI:** 10.1002/hpm.3939

**Published:** 2025-04-21

**Authors:** Sarah Buckingham, June Ann De Vera, Lorraine Faeldon, Bridie Kent, Angela Logan, Aira Ong, Nena Marie Santos, Paula Melizza Valera, Jonathan Marsden

**Affiliations:** ^1^ Faculty of Health University of Plymouth Plymouth UK; ^2^ De La Salle Medical and Health Sciences Institute College of Medicine Dasmariñas Philippines; ^3^ De La Salle University ‐ Evelyn D. Ang Institute of Biomedical Engineering and Health Technologies Manila Philippines; ^4^ Royal Devon University Healthcare NHS Foundation Trust Exeter UK

**Keywords:** healthcare policy, interviews, low‐middle income countries, Philippines, stroke care, stroke rehabilitation, survey

## Abstract

**Objectives:**

This study aimed to illustrate the state of stroke care and rehabilitation in the Philippines through the perspectives of local government officials, policymakers, and organisational leaders. It sought to identify challenges, opportunities, and recommendations for improving stroke policies and services across different administrative levels.

**Methods:**

Mixed‐methods approach involving a structured survey of 131 local government officials and in‐depth interviews with eight key stakeholders. Survey participants included Department of Health (DoH) officials, local chief executives, policymakers, Local Government Unit (LGU) employees, and representatives from non‐government agencies. Interviewees comprised leads and managers from the DoH and representatives from organisations including the Philippine Academy of Rehabilitation Medicine (PARM), Physicians for Peace Philippines, and the Philippine Council for Health Research and Development (PCHRD). Quantitative survey data were analysed using descriptive statistics and qualitative interview data were thematically analysed, then the two types of data were triangulated and organised by theme.

**Results:**

Findings revealed significant gaps in funding, healthcare infrastructure, and policy implementation. Challenges included inadequate facilities, lack of qualified staff, financial barriers, and regional disparities in service provision. Survey and interview participants emphasised the need for increased government support, comprehensive policies, and community‐based rehabilitation (CBR) programmes. Improving stroke survivors' quality of life was ranked as the most critical aspect of rehabilitation programmes.

**Conclusions:**

The study highlights the critical need for more equitable and accessible stroke care and rehabilitation in the Philippines. This can be facilitated by sustained government support, inter‐agency collaboration, community engagement, and the implementation of holistic, evidence‐based, and cost‐effective CBR initiatives.

**Summary**

- Current Philippines stroke policies are generic and limited to clinical care
- There are challenges in implementing local policies and Universal Health Care
- Policymakers are supportive of community‐based rehabilitation
- A multi‐sector, holistic, prevention‐ and education‐focused approach is needed.

## 1 Introduction

Stroke is a leading cause of disability and mortality worldwide, affecting at least 12.2 million annually. It is estimated that 143.2 million years of healthy life are lost each year from deaths and disability due to stroke [1]. Furthermore, the incidence of stroke is believed to continue to increase due to the rising number of non‐communicable diseases, such as hypertension and diabetes, which remain challenging to address at a population level [2]. This burden poses significant implications for many health systems in general, particularly and disproportionately affecting low‐ and middle‐income countries (LMICs), like the Philippines [3].

A recent scoping review identified inequitable stroke care and rehabilitation in the Philippines, with the private sector dominating services and costs borne out‐of‐pocket by people with stroke and their families [4]. A wide gap exists between the high burden of stroke in the Philippines and the availability of stroke services; this includes a lack of emergency medical services, stroke ready hospitals (facilities and workforce), acute treatments (e.g., thrombectomy and thrombolysis), and rehabilitation (hospital and home‐based) [5]. Such gaps are seen in other LMICs, reflecting challenges in implementing stroke services in resource‐constrained countries [6, 7].

In the Philippines, the enactment of the Universal Health Care (UHC) Act in 2019 [8] marked a significant milestone in the country's health sector, which aims to ensure that all Filipinos have equitable access to quality and affordable healthcare services. The law mandates the automatic enrolment of all citizens in the National Health Insurance Programme (NHIP) managed by the Philippine Health Insurance Corporation (PhilHealth). It emphasises the creation of city‐wide or provincial‐wide Health Care Provider Networks (HCPNs) and comprehensive life‐stage health services, which are integral for providing coordinated and integrated health services at different levels of healthcare facilities. Despite the enactment of UHC and the expansion of PhilHealth coverage, massive gaps in the coordination and comprehensive care still exist, particularly in post‐acute and long‐term care, and indirect costs such as transportation and caregiving [9]. These gaps reflect the challenges seen in stroke care. A better understanding of the current issues in stroke care can therefore inform the equitable delivery of services within the UHC scheme in the future. The UHC Act could facilitate ease of navigation, clearer referral processes and better continuity of care from acute treatment to rehabilitation [8].

Stroke rehabilitation, an essential part of stroke care, provides an opportunity to promote recovery, restore function, maximise an individual's ability to perform activities of daily living, and allow continued participation in various aspects of life [10, 11, 12]. The early initiation and continuity of effective stroke rehabilitation is essential for improving the quality of life of stroke survivors, depending on the extent of their stroke lesions, and reducing the long‐term burden on healthcare systems. However, the current state of stroke care and rehabilitation in the Philippines remains under‐researched, with substantial gaps in policy, funding, and service delivery [3]. Studies have shown that the lack of specific guidelines and financial support limit the implementation of effective rehabilitation programmes [13]. The role of government policies and the need for integrated care models have been highlighted as critical factors in improving stroke rehabilitation outcomes [14].

The need to establish cost‐effective stroke rehabilitation programmes has been recommended in the Philippines [5] and more generally in LMICs [7]. Community‐based rehabilitation (CBR) has been recognized as a promising approach to enhance access to care and reduce inequities in healthcare delivery. CBR initiatives have been successful in other LMICs by leveraging local resources and promoting community involvement [15]. However, the effectiveness and perceptions of CBR in the context of stroke rehabilitation in the Philippines remain largely unexplored.

It is crucial to consult with all stakeholders to improve our understanding of the issues and explore different perspectives and needs, to inform working towards more equitable and effective stroke care and rehabilitation services in the Philippines. The limited number of studies in this field have primarily focused on data gathered by or from healthcare professionals working in clinical settings [4, 5], and there is little or no evidence from the perspectives of those involved in policy development and implementation. In the TULAY (Tulong, Ugnayan ng Lingap At gabaY) project, a multi‐sector survey and semi‐structured interviews enabled exploration of the perspectives of various stakeholders: people with stroke, their household carers, healthcare providers, government officials, policymakers, and relevant organisational leaders. This paper focuses on the perspectives of government officials, policymakers, and relevant organisational leaders. Through their involvement in the planning and delivery of stroke services, these stakeholders provide a vital understanding of the facilitators and barriers to future policy decisions. The aims of this study were to advance understanding of stroke care at the macro (national) level, including identifying challenges, opportunities, priorities, perceptions of CBR, and recommendations for improving stroke policies and services across different administrative levels.

## 2 Methods

### 2.1 Overview of Methods

This study employed a cross‐sectional design, utilising a mixed methods approach with concurrent data collection (i.e., survey and interviews conducted simultaneously). This approach was used to provide a more comprehensive picture of the perspectives of the participants. The GRAMMS checklist for mixed methods studies in health services research was followed [16] (Supporting Information S1).

### 2.2 Survey Methods

#### 2.2.1 Design and Sampling

A structured survey gathered quantitative data from government officials involved in healthcare policy development or execution, regardless of their previous involvement in stroke‐related initiatives. A stratified sampling method was employed to ensure that participants were representative of different geographic regions (National Capital Region [NCR], Region IV‐A [CALABARZON], Region VI [Western Visayas], Region VII [Central Visayas] and Region X [Northern Mindanao]), a range of income generation brackets, various government levels (provincial, municipal and barangay), and both urban and rural areas. The aim of this was to provide a balanced distribution of perspectives on stroke care and rehabilitation, minimising sampling bias and enhancing the generalisability of the findings [17, 18]. Participants within the selected areas were identified through governmental directories and official lists.

#### 2.2.2 Instrument

The survey instrument was a self‐report questionnaire, with questions informed by the findings of a scoping review that mapped available literature on stroke services in the Philippines and identified gaps and potential solutions. The questionnaire was collaboratively designed by healthcare and public health professionals and researchers, within and external to the TULAY project team. A combination of multiple‐choice, ranking and open‐ended (free text) questions was used to capture demographic information, roles and responsibilities, involvement in healthcare budget planning, and perspectives on stroke care and rehabilitation (Supporting Information S2). Specific sections included:

*Section* 1: Participant's role, level of government, and branch of government.

*Section* 2: Involvement in healthcare budget planning and decision‐making.

*Section* 3: Prioritisation of areas for improvement in stroke care and rehabilitation.

*Section* 4: Perceptions of community‐based rehabilitation (CBR) programmes.

The questionnaire was available in two languages, English and Tagalog. Pilot testing of both language versions was done within the research group before distributing it more widely. The questionnaire took approximately 15 min to complete.

#### 2.2.3 Data Collection

The questionnaire was available in two formats; online via Jisc [19] and offline (paper). This was for participants' convenience and to increase reach, for example in areas with limited internet connections or where there was difficulty reaching respondents.

The mayor in each selected municipality was informed about the study and invited to participate via letter or e‐mail; they were provided with an information sheet, consent form, link to the online questionnaire, and a copy of the paper questionnaire (where needed). Paper questionnaires were disseminated by research assistants and field enumerators and later collected for online data entry by the research team.

The mayors were gatekeepers for the Barangay Captains, local officials and government employees within the cities, municipalities and targeted barangays. They provided formal endorsement to access the area and to permit the research team to contact the additional participants regarding participation in the survey.

Follow‐up reminders (e‐mail, phone call or a visit to the office) were given to maximise response rates. The anonymity of responses was maintained to encourage candid participation. Data collection began in November 2023 and concluded in May 2024.

#### 2.2.4 Data Analysis

Following data cleaning and removal of duplicates, quantitative data from the survey were analysed using descriptive statistics, including means, percentages, and frequency distributions. These analyses provided an overview of the current state of stroke care and rehabilitation from the perspective of local government officials. The programme used in analysis was IBM SPSS Statistics software version 29 [20].

To calculate rankings based on survey responses, the weighted ranking method is used with the formula below. The item with the highest score is considered the highest priority.

$$\text{Score}=\left[\left(R_{1}\times n\right)+\left(R_{2}\times \left(n-1\right)\right)+\text{…}+\left(R_{n}\times 1\right)\right]$$

Where:

*n* = number of items to rank.

*R*_1_, *R*_2_, *R*_n =_ are the counts of how often each item was ranked 1st, 2nd, …, *n*th.

Free text survey responses were analysed using qualitative thematic analysis [21, 22].

### 2.3 Interview Methods

#### 2.3.1 Design and Sampling

Semi‐structured interviews were used to explore current and future plans for community rehabilitation provision and stroke services from DoH officials, health officers, and leads of relevant organizations including the Philippine Academy of Rehabilitation Medicine (PARM) and the Philippine Council for Health Research and Development (PCHRD). A purposive sample of participants was identified from government and online registries. Selected participants were provided with an invitation letter (via e‐mail or delivered in person) with an information sheet and consent form that was completed prior to the interview.

#### 2.3.2 Interview Instrument

An interview schedule developed by the research team was followed for the semi‐structured interviews. This included a topic guide (Supporting Information S3) covering four major topics:

1. Demographics and interviewee roles
2. Structure and organization of stroke care
3. Perceived changes to stroke care, barriers and facilitators
4. Financial aspects of stroke care which was divided into acute care, rehabilitation and stroke care

#### 2.3.3 Data Collection

The interviews were carried out online (via Zoom) by researchers in the Philippines. One of five research leads conducted the interview while a research assistant made observations and took field notes. All researchers had previous experience of conducting interviews in a health research setting. Interviews were audio‐recorded with participants' consent, transcribed verbatim, and translated into English where necessary. Interviews were conducted between January and March 2024.

#### 2.3.4 Data Analysis

Data were analysed using thematic analysis [21, 22]. This was conducted by two UK researchers (SB and AO) and two Philippines researchers (NS and Marie Cieli Aragon) with training and experience in qualitative research. Following familiarisation with the transcripts and field notes, the researchers independently coded the data before meeting to compare responses and to agree on the coding scheme. There was high agreement between the researchers' coding, and regular meetings were held to review and refine the identified themes. Both inductive and deductive analysis were used, the former identifying themes in the data, and the latter drawing on concepts within the topic guides. NVivo version 14 [23], Microsoft Word and Excel were used to support analysis, with the use of manual coding, highlighting of key themes and quotations, and reflective memos.

Various measures were taken to enhance rigour and to ensure quality of the interview study [24]. These included use of an interview schedule with clear definitions of terms used; meticulous record‐keeping with a clear audit trail to source data; critical reflection throughout data collection and analysis; and input from multiple researchers with triangulation and sharing of perspectives.

#### 2.3.5 Data Integration

As a mixed methods study, the quantitative and qualitative data were integrated following analysis, during the interpretation phase, and discussed with all co‐authors. Combining the two sources of data enabled expansion (where interviews expanded on survey findings), and complementarity, that is exploring related but different facets of a phenomenon to yield an enriched understanding [25]. The findings were triangulated and organised by theme.

## 3 Results

### 3.1 Survey Participant Characteristics (Demography, Roles and Government Level)

**FIGURE 1:**
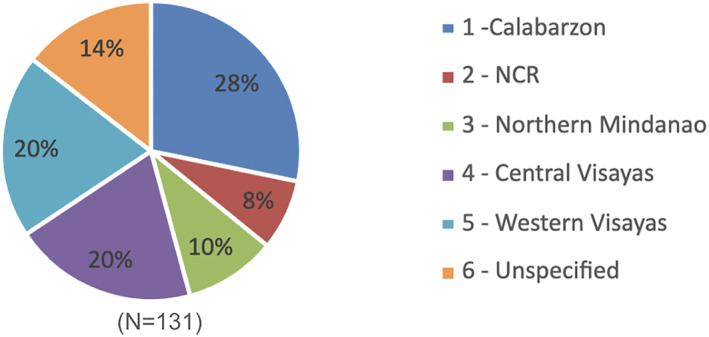
Regional distribution of survey respondents.

One hundred and thirty‐one survey responses were received from local government officials and employees across the regions in the sampling strategy (Figure 1). Participants were categorised based on their roles within the government (Table 1). For the distribution of respondents across different branches of government, 36% (*n* = 48) of respondents worked in the executive branch, 18% (*n* = 24) in the legislative branch, 6% (*n* = 8) had dual roles, and 35% (*n* = 47) were involved in local office implementation. In terms of various administrative levels in the Philippines, the majority (96.18%, *n* = 126) were employed at the municipal/city level, far outnumbering those working at the national (2.3%, *n* = 3), regional (0.8%, *n* = 1), and provincial levels (0.8%, *n* = 1). Finally, a significant 76.3% (*n* = 100) of the respondents were involved in healthcare budget planning.

**TABLE 1:** Distribution of survey respondents by role.

Role in government	Frequency	Percent (%)
Department of Health Official	4	3.05
Local Chief Executive (e.g., Mayor, Governor)	18	13.74
Local policymaker or councillor	9	6.87
Local Government Unit Employee (MHO, PSWDO, PDAO etc.)	99	75.57
National Council for disability Affairs (Under the Department of Social Welfare)	1	0.76
Total	131	100

### 3.2 Interview Participant Characteristics

Twenty‐three government officials and organisational leaders were invited to participate in an interview; of these, eight agreed to take part. Seven interviews were conducted with the eight participants (one was a joint interview with two participants). Participants included leads and managers from the DoH (*n* = 5) and representatives of other relevant organisations (*n* = 3) including PARM, Physicians for Peace Philippines, and the PCHRD. Tenure within the current organisation ranged from 2 years to 35 years. Five interviewees were female and three were male. Most interviews (*n* = 5) were conducted in English or English/Tagalog, one was in Tagalog, and one was in Bisaya. The interview duration ranged from 34 to 90 min.

### 3.3 Summary of Themes

Four themes were identified from the survey and interviews, each with several sub‐themes. These are described below and summarised in Table 2.

**TABLE 2:** Interview themes and sub‐themes.

Theme	Sub‐theme
1. Existing stroke care and rehabilitation: Policies, governance and funding	Generic nature of policies and guidelines Adopting a healthcare policy Funding of healthcare
2. Challenges and inequities in stroke care	Challenges at all stages of the stroke pathway Lack of healthcare facilities and workforce Lack of accurate stroke data Financial challenges Variability in services
3. Community‐based rehabilitation (CBR)	Perceived importance of CBR Level of interest in CBR Aspirations for CBR Concerns related to CBR
4. Improvements and recommendations for stroke care and rehabilitation	Inter‐agency collaboration and local leadership Holistic, individualised approach Technological solutions Building the evidence base Preventive care and educating communities Other recommendations

#### 3.3.1 Existing Stroke Care and Rehabilitation: Policies, Governance and Funding

The survey and interviews provided information on current healthcare policies and how they are adopted, and confirmed the challenging political and financial landscape of stroke care and rehabilitation in the Philippines.

##### 3.3.1.1 Generic Nature of Policies and Guidelines

The interviews revealed that current guidelines and government policies are generic rather than specific to stroke. There are organisational guidelines, which are not mandated by the government, although they are disseminated to the general population and the health ministry:

It's more generalised rather than specific for stroke. There are guidelines that are set, that are released by the Stroke Society of the Philippines. The Philippine Academy of Rehabilitation Medicine has CPGs [Clinical Practice Guidelines] on stroke as well. (But), again, those are not policy of the government.

(Interview 007)

The interviewees alluded to the National Framework for acute stroke management [26] but recognised that guidance on rehabilitation and social care is limited. They emphasised the need for more comprehensive policies at national and regional levels that should be implemented at the Local Government Unit (LGU) level, that is for provinces, cities and municipalities.

##### 3.3.1.2 Adopting a Healthcare Policy

Survey respondents (*n* = 131) ranked the types of information deemed most useful before adopting a healthcare policy (Table 3). Respondents ranked knowledge of the impact of the condition on a person's function and quality of life as the most important information to them when deciding to adopt a healthcare policy.

**TABLE 3:** Ranking of useful information before adopting a healthcare policy (*n* = 131).

Item	Score	Final rank
Knowledge of the impact of the condition on a person's function and quality of life	376	1
Knowledge of how many people have the condition in your area	338	2
Knowledge of the impact of the condition on a person's family in terms of ability to continue working	312	3
Knowledge of potential healthcare resources and costs	302	4

The open‐text responses to the question, ‘What information is useful to you before adopting a healthcare policy?’ highlighted several key considerations beyond the predefined options. Respondents emphasised the importance of understanding potential government restrictions, particularly in the procurement of medicines and medical supplies for Programmes, Projects and Activities (PPAs) if they are non‐standard. Cultural factors, such as the culture of people living in a certain area, were also mentioned as crucial for policy adoption. Additionally, health information, including mortality data and the awareness of policymakers from the national to the barangay level, as well as the efficiency of the Health Information System, were seen as critical. Local ordinances providing budgetary assistance for the medical concerns of indigents and the need for community participation, along with inter‐agency and intra‐agency coordination, were identified as vital components. Sustainability of healthcare policy was also a significant concern, as were local health statistics and the annual investment review. Finally, the readiness of potential partners and ensuring that the community acknowledges and accepts the existing health issues before becoming agents of change were noted as important factors in the decision‐making process.

**FIGURE 2:**
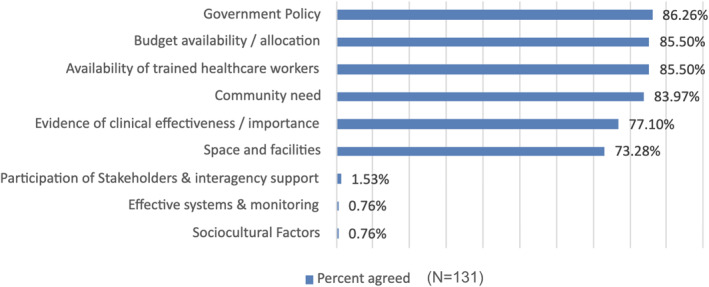
Key factors for adopting a new health policy.

When adopting a new health policy, several key factors must be considered. According to the survey respondents, the most critical factors are alignment with government policy, followed by budget availability, availability of trained healthcare workers, community need, evidence of clinical effectiveness, and space and facilities (Figure 2). Lesser considerations include the participation of stakeholders and interagency support, effective systems and monitoring, and sociocultural factors.

In addition to the factors ranked, the free text responses included the following: (1) Government mandates play a crucial role, with respondents noting that their ‘hands are tied’ if it is a national‐level directive, though community needs remain a significant consideration in the absence of such mandates. (2) Political, social, and cultural factors, as well as workforce and resource availability, are essential in determining the feasibility of implementation. (3) Inter‐agency participation and community support are viewed as vital for successful policy integration. (4) The role of national and local government support, including the presence of effective support systems, is also critical. (5) Effective system for monitoring programmes, and (6) collaboration with stakeholders were all seen as important elements for achieving broad‐based buy‐in and cooperation.

In ranking factors influencing the adoption of a new ordinance (Table 4), local voices emerged as the most important, followed by government policies. Local platforms were considered the least significant.

**TABLE 4:** Ranking of factors influencing the adoption a new ordinance (*n* = 131).

Item	Score	Final rank
Local people's voice	298	1
Government policy	279	2
Policy mentioned in your local platform	215	3

*Note:* An ordinance is a localised policy created by a local government when adopting a national policy.

##### 3.3.1.3 Funding of Healthcare

Various forms of financial support for people with stroke are available including PhilHealth, the Philippine Charity Sweepstakes Office (PCSO) and the Department of Social Welfare and Development (DSWD). However, some interviewees talked about the limitations of PhilHealth, which excludes rehabilitation costs, and indirect costs associated with therapy such as travel expenses:

As far as I know there's a stroke‐, it's covered by PhilHealth… there is a certain amount. I'm not sure how much it is but the rehabilitation of the stroke is not included.

(Interview 007)

[The] PhilHealth package only solves the direct costs, meaning the direct cost of the physician, therapy sessions, but it does not respond to the indirect cost ‐ of the travel, the bringing of the patient from the house to the facility, from the house to the rehab session.

(Interview 006)

#### 3.3.2 Challenges and Inequities in Stroke Care and Rehabilitation

Challenges in stroke care and rehabilitation at multiple levels were reported in the interviews. The greatest challenges were recognised in rural and isolated regions.

##### 3.3.2.1 Challenges at all Stages of the Stroke Pathway

All interviewees identified challenges at all stages of the stroke pathway. For example, in acute stroke care, interviewees talked about the ‘*golden period*’ for recovery, where treatment needs to be started as soon as possible but the availability of this is ‘*facility‐based*’. Similarly, early rehabilitation was perceived as critical but inconsistently available, and home rehabilitation is extremely limited.

Formal social care services and care homes are limited. Social care is primarily handled through general programmes for Persons with Disabilities (PWD) or senior citizens, with no specific focus on post‐stroke care. Long‐term care is typically provided by families, but there are concerns related to family members' competency in providing this care, for example some families may be overprotective of the person with stroke:

The family takes the burden of care in terms of the Filipino… it's a two‐edged sword, we are too nice. We take care of our elderlies, we take care of our sick, but sometimes we over care. Sometimes the patient [is] able to walk… you won’t have them walk because they are sick or they have a disability.

(Interview 007)

##### 3.3.2.2 Lack of Healthcare Facilities and Workforce

In addition to a lack of healthcare facilities, there is a lack of qualified staff to deliver stroke care and rehabilitation. The interviewees talked about the limited availability of services including preventive care, primary care and rehabilitation, referral system issues, low numbers of staff specialising in neurology and rehabilitation, and high staff turnover with many staff moving abroad. These issues are widespread, but most pronounced in rural areas:

… actually, the reality is at the rural level, in most of the barangays in the Philippines, there's not even a doctor. They're fortunate enough to have a nurse or maybe a midwife…

(Interview 004)

##### 3.3.2.3 Lack of Accurate Stroke Data

Existing stroke data from local to national levels were perceived as incomplete and inaccurate, which makes policymaking more difficult.

The current available data in the Department of Health is messy.

(Interview 002)

##### 3.3.2.4 Financial Challenges

Financial challenges were a central theme in all the interviews, experienced at various levels and from acute care to long‐term rehabilitation. In addition to recognising the limitations of existing government and organisational funding, the interviewees were sympathetic to the financial barriers experienced by individuals which include significant costs for treatment, travel, and loss of income for the person with stroke and their companions:

… they don't have the money to go to the facility, they are afraid that when they go they just end up spending everything they have…

(Interview 004)

One interviewee gave an estimate of the cost of a single rehabilitation session, equivalent to approximately 2 days' income. Another talked about people having to make a choice between seeking medical treatment and day‐to‐day survival:

Food or medicine? Food or therapy, where do you go? So, it will go ‐ of course, you’ll go to the basic necessity to survive.

(Interview 007)

People from low‐income backgrounds and those living in poorer, more rural areas were deemed to experience the greatest financial barriers to accessing and receiving care and rehabilitation.

##### 3.3.2.5 Variability in Services

In addition to inequities in stroke care and rehabilitation at the individual level, the interviewees talked about the differences between urban and rural areas, public and private hospitals, and regional disparities.

In public healthcare settings, not all government hospitals have rehabilitation departments:

… it depends if you're in the public or private setting… not all government hospitals have rehabilitation doctors.

(Interview 007)

Private hospitals are generally better equipped, but the process often depends on referrals from primary neurologists.

Quality and access to care vary by region; this is influenced by resources and the level of specialisation of the hospital (i.e., level 1, 2 or 3):

… especially if you’re in the province, it’s not every day there’s a rehab doctor there… it really depends on the region… depends on the budget.

(Interview 007)

The interviewees reported that urban and better‐funded areas offer more comprehensive services, with most rehabilitation centres situated in specialised and private hospitals in urban centres.

#### 3.3.3 Community‐Based Rehabilitation (CBR)

The survey and interview participants were generally supportive of CBR and held positive perceptions, although one concern was mentioned in the interviews.

##### 3.3.3.1 Perceived Importance of CBR

In relation to other policy areas, healthcare rehabilitation was ranked third by the survey respondents, after healthcare prevention and food security (Table 5).

**TABLE 5:** Perceived local importance ranking.

Item	Score	Final rank
Healthcare prevention	759	1
Food security	624	2
Healthcare rehabilitation after disease or injury	541	3
Economic development	522	4
Disaster mitigation	467	5
Peace and order	463	6
Infrastructure projects	361	7

##### 3.3.3.2 Level of Interest in CBR

**FIGURE 3:**
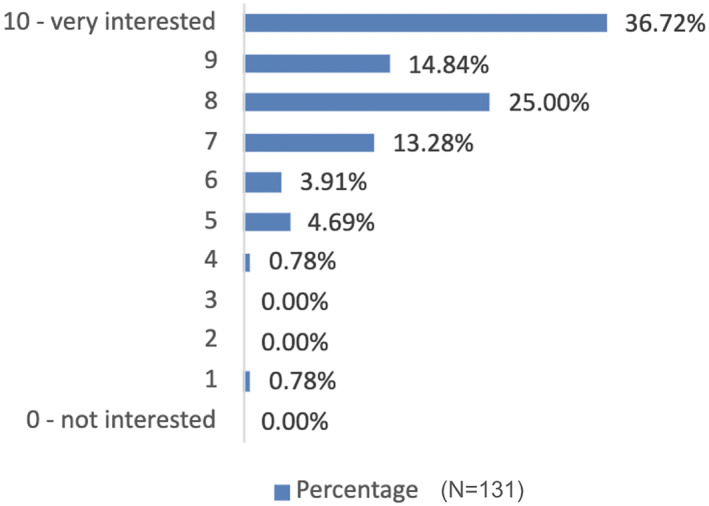
Distribution of interest in CBR projects.

A high level of interest in CBR was found in the survey (Figure 3). The highest percentage of respondents (36.72%) rated their level of interest in CBR projects as 10 (very interested), with the majority (89.84%) rating their interest as 7 or above.

##### 3.3.3.3 Aspirations for CBR

Interviewees talked about the current lack of CBR in the Philippines, but spoke positively about its potential for stroke (and other conditions). It was perceived as a way to reduce inequities and improve access to care, and some were already working to promote this within their organisations:

We don’t really have community‐based care… we are still in the birthing pains.

(Interview 003)

What we really want is for community‐based rehabilitation for those with stroke to be part of the primary healthcare network… what I feel is that if this will be implemented, access will no longer be a problem… So that is actually one policy which we are really pushing for now and we are very happy that DoH [Department of Health] created the task force on community‐based rehabilitation.

(Interview 006)

Survey respondents were asked to rank several areas of improvement in CBR programmes in terms of importance, and the highest ranking was improving people's quality of life and happiness, which directly influences overall well‐being and satisfaction (Table 6).

**TABLE 6:** Ranking importance of improvement areas in CBR programmes.

Item	Score	Final rank
Improvement in people's quality of life and happiness	449	1
Improvement in ability to do things	341	2
Ability to return to work	304	3
Decrease in burden for carers and family members	274	4

##### 3.3.3.4 Concerns Related to CBR

Although there were no negative perceptions of community‐based rehabilitation per se, one interviewee talked about self‐management at home or in the community being viewed by health professionals as a potential barrier to healthcare. This is because in the Philippines culture, people tend to manage themselves first and only seek help when their condition becomes severe, untreatable, or unbearable:

I think that's also a barrier because in the Philippines, most patients, particularly in the rural areas, they do not consult immediately a doctor. They tend to manage themselves first and only when they cannot bear it anymore or it becomes really severe, that's the only time they go to a doctor.

(Interview 004)

Therefore, there may be a need to change the perceptions of self‐management and community‐based care in general.

#### 3.3.4 Improvements and Recommendations for Stroke Care and Rehabilitation

Interviewees and survey respondents made several recommendations to improve stroke care and rehabilitation.

##### 3.3.4.1 Inter‐Agency Collaboration and Local Leadership

The need for inter‐agency cooperation and collaboration was a common theme and perceived as essential in implementing care and rehabilitation programmes. The interviews highlighted the importance of collaboration within and between government agencies including the DoH, DSWD, LGUs, Rural Health Units (RHUs), and Technical Education and Skills Development Authority (TESDA), and non‐government organisations.

They should not work in silos; they should work together… they should talk to each other. Otherwise, you will not be able to implement community initiatives.

(Interview 006)

Local (LGU) ownership and implementation of policies was seen as vital. Health should be more widely recognised as a priority, which is not always the case at present:

Healthcare services are more abstract and harder to see, which affects votes… some LGUs are not supportive of health, seeing it as DoH’s work alone… but in reality, health is a collaborative effort. DoH’s role is to augment health services and the LGU should lead, but some LCEs [Local Chief Executives] do not see it this way.

(Interview 001)

The need for continuous support and commitment from local leaders was viewed as important for the sustainability of initiatives:

… we actually ensure that when we leave the community, we already have an established LGU or a partner that will continue the services we started… they would have ownership and render it sustainable.

(Interview 006)

Working with community groups and support groups was also perceived as key by survey and interview participants; this ensures that policies are person‐centred and meet the needs of people with stroke and other disabilities. However, none of the interviewees were aware of any specific community programmes or support groups for stroke, as there are for other conditions like diabetes.

##### 3.3.4.2 Holistic, Individualised Approach

A holistic approach to stroke care that meets the needs of individuals was advocated by the interviewees. Depending on individual needs, this should include medical, psychological, social, financial and return to work support. Reintegration into the community was perceived as highly important, as was a need to strengthen or expand the ‘*social safety net*’. Extending community‐based programmes beyond stroke to other chronic conditions was also recommended:

It is possible that not only stroke patients need rehabilitation… you can take a holistic approach or integrated approach.

(Interview 003)

##### 3.3.4.3 Technological Solutions

Utilising available technologies and trialling new technologies was recommended; interviewees mentioned assistive devices, mobile rehabilitation workshops, smartphone apps, and telerehabilitation. These were trialled in certain areas during the COVID pandemic and were an effective way of improving accessibility to rehabilitation for people living in remote and rural areas. However, cost was recognised as a barrier to more advanced technologies such as robotics.

##### 3.3.4.4 Building the Evidence Base

All of the interviewees talked about the importance of research and implementation science to ensure that policies are evidence‐based, applicable to real‐world settings and sustainable. For example:

… you need to develop evidence… because you're doing this research… if you're not able to develop evidence, it will not become a policy.

(Interview 006)

At the end of the day, we want the Filipinos to benefit from research and the only way to do that is of course to make sure that it's supported by very strong implementation science.

(Interview 004)

The need for more accurate stroke data to inform decision‐making and improve care strategies was emphasised. For example, a national registry or directory of people with stroke to survey their needs was seen as an important next step.

##### 3.3.4.5 Preventive Care and Educating Communities

Interviewees talked about the need for a ‘*paradigm shift*’ in the Philippines, from curative to preventive healthcare. This includes improving the screening and management of risk factors at the RHU level to prevent stroke. This will benefit communities and reduce pressure on health services.

… I think we have to invest more on the prevention side… we need continuous health promotion among Filipinos and the family taking care of stroke patients.

(Interview 005)

The need to improve health literacy was emphasised; health education, health promotion and raising awareness are currently limited:

… health literacy on how to take care of ourselves already when we are at risk and sick… you still don't see a lot of messages [about it] around.

(Interview 002)

Interviewees reported that many people do not know what a stroke is, the different types of stroke, its causes, nor its consequences, and there is a lack of awareness of physical rehabilitation and its benefits (the term ‘rehabilitation’ tends to be associated with drug abuse and mental health). Knowledge of stroke and the nature and benefits of rehabilitation need to be improved:

… Rehabilitation is left behind because they’ll say, ‘It’s just exercise anyway, it’s just moving this and that.’ And then, the effect or the result, what you want to happen ‐ moving the hand, for it to function ‐ it doesn’t occur in one to two sessions. So, it will entail a lot of frustration, patience and dedication.

(Interview 007)

There was a consensus that education and training programmes should be directed towards community health workers (including barangay health workers, nurses and midwives) as well as people with stroke and their families, and the wider community:

You have to educate not only the patient, but also, the caregiver and the society in general.

(Interview 007)

The interviewees reported that barangay health workers are best placed to identify people with stroke, but they should not be overburdened and need support from others:

It’s the barangay health workers that can help patients… Number one is determining where these patients are, who these patients are and what are their needs. So, at the community, if you train the barangay health worker they will see them faster.

(Interview 007)

They’re doing so much already so they’re tired already. They do vaccinations, they take care of the health and wellness programme. They’ll say, ‘Here we go again, there’s training again.’ Our target for example for assistive tech and rehab are not actually the barangay health workers anymore… they're so overworked and sometimes when the mayor changes or whoever would be the person in charge, the barangay health workers that you trained also changes or leaves and so, you train again.

(Interview 006)

It was suggested that family caregivers should be encouraged not to be too overprotective, and to support the person with stroke to be independent rather than doing everything for them. Education and training programmes should be mindful of this.

##### 3.3.4.6 Other Recommendations

The need for increased government funding to improve stroke care infrastructure and services was recognised. This includes expanding PhilHealth coverage to include comprehensive stroke rehabilitation, equitable distribution of resources to prioritise underserved areas, and providing subsidies to alleviate the financial burden on people with stroke and their families. The need to integrate services at provincial and city levels when implementing UHC was recognised:

At the heart of the universal healthcare is the primary healthcare, meaning health services should be available where the people are… maybe rehabilitation for stroke patients is not yet at the primary healthcare level.

(Interview 004)

The need to further develop and standardise national clinical guidelines, and the importance of early rehabilitation and continuity of care were also highlighted.

## 4 Discussion

Overall, there was good triangulation of themes between survey and interview data. Key themes included the challenging political and financial landscape of stroke care and rehabilitation; challenges and variations in care at multiple levels; support for CBR; and the need for a multi‐sector, holistic, prevention‐ and education‐focused approach.

The results demonstrate the vast differences and inequitable distribution of stroke care, services, facilities, and trained staff, and illustrate the critical shortcomings of current stroke public policies. Whilst there is a National Policy Framework on the Prevention, Control and Management of Acute Stroke in the Philippines [26] and health professional‐led guidelines in acute stroke care disseminated with national government agencies, there are clear limitations in long‐term preventative and rehabilitative care, when patients are back in their respective communities [27].

The findings mirror similar challenging experiences in stroke care systems across LMICs [6, 7]. Many LMICs are facing difficulties in absorbing the demands in care and service delivery, of a public health concern that is projected to increase across age groups and sexes [28, 29]. Evidence suggests that stroke care in LMICs, albeit heterogeneous and difficult to compare, often faces fragmentation, and is compounded by the challenges of implementing evidence‐based interventions in local practice [30]. This highlights the gaps in adoption and feasibility in low‐resourced settings, as most studies come from high income countries [30, 31]. Whilst there are universal minimum stroke care elements (e.g., CT scans) [32], robust and organised stroke care systems need contextualisation and understanding of a country's socio‐cultural, geographic and financial disparities [33, 34]. The Philippine stroke care system is within a context of predominantly private sector owned health facilities, with public health services dependent on local government, which further adds to the challenges. Many of these issues are recognised in the World Health Organisation's Rehabilitation 2030 call for action [35].

Haemorrhagic and ischaemic strokes are in the top 10 health conditions that contribute to 40% of the Philippine Disability Adjusted Life Years (DALYs), and are recommended priorities for service coverage by PhilHealth and the DoH [36]. The DoH has released their Health Facility Development Plan that outlines infrastructure, service capacities and equipment investments [37]. Further, the health ministry has designated some of their retained tertiary facilities as speciality centres for cardiovascular care and brain and spine care [38]. PhilHealth has shown some efforts in covering stroke‐related care. In 2023, there was an increase in acute stroke care coverage, but current case rates only include minimal hospital admission standards [39]. Recently, PhilHealth published their policy to cover physical and rehabilitation services, and mobility assistive devices for adults [40]. Additionally, a law on mandatory PhilHealth coverage for registered persons with disabilities to reduce out‐of‐pocket and catastrophic costs for secondary preventative and rehabilitative care exists, but its implementation and uptake remain uncertain [41].

The results also highlight the paucity of stroke‐related data, overall. It is crucial to have accurate, localised data to guide policies, programmes and other interventions that are appropriate for the varied Philippine contexts. This can be addressed, at the very least, by formalising and taking concrete steps in developing the formation of a framework of the hospital‐based National Stroke Registry [26]. In 2020, the Philippine Neurological Association (PNA) established the PNA One Database‐Stroke (PNA1DB‐Stroke), a multi‐centre observational study involving 11 accredited neurology residency training institutions in the Philippines. This is a significant initiative launched in 2021 to systematically collect and analyse stroke‐related data, including patient demographics, treatment outcomes, and resource utilization [42, 43].

Another important development is the implementation of the Hub‐and‐Spoke Stroke Care System in 2021; it aims to address disparities in access to stroke care, especially in underserved areas in the Philippines. In this model, primary care facilities (spokes) are connected to specialised stroke centres (hubs) through a coordinated network. Early results from pilot implementations have shown promising outcomes, including reduced treatment delays and improved patient outcomes [44]. Community‐based rehabilitation has been nationally recognised as a key element in promoting comprehensive and accessible care to persons with disabilities [45], yet it is unknown to what extent this has been established within the various LGUs. CBR has been generally provided by non‐government organisations, to mitigate the gaps in services, but its continuity and outcomes, specifically in stroke‐related conditions, have yet to be determined.

At present, the National Council on Disability Affairs has begun initiating Community Based Inclusive Development (CBID) programmes in selected areas in the country which aims to bring rehabilitation services closer to the people with stroke and other disabilities in the community. There is, however, a need for support through more policies or ordinances in the LGU level for the implementation of CBID. CBID has different components, namely Health, Education, Livelihood, Social, Empowerment, and Environment. The CBID Health component focuses more on health promotion, prevention, medical services, rehabilitation services and provision of assistive devices [46, 47]. Once fully implemented, people with stroke and the community can benefit from all these different components. With ordinances or policies supporting CBID, centres for Rehabilitation Services may be established by the LGUs, and plantilla (regular full‐time employment) positions for healthcare workers may be provided, which will improve the delivery of stroke rehabilitation services. Through CBID, people with stroke and their families can also benefit from training and participate in the different programmes for social skills development, vocational, recreation, leisure, sports and empowerment. Furthermore, these efforts can best address improving stroke education and stroke health literacy, addressing practices towards secondary prevention.

The Philippine public health system is devolved, decentralising the implementation of health programmes and services under the stewardship and administration of provincial, city, and municipal local governments. However, Philippine healthcare devolution has been perceived as challenging due to the disconnect between national government policies and the varied capacities of local governments to manage health systems and effectively implement health programmes. This is most felt in geographically isolated and disadvantaged areas [48, 49]. The survey and interview findings suggest that whilst there is intention for uptake of policies and implementation of new programmes, the capacity for readiness in resource planning and mobilisation, and service delivery for stroke care of local governments remain in question. Although initiatives such as the Hub‐and‐Spoke Stroke Care Systems and CBID programmes hold promise, challenges remain in scaling up these efforts, particularly in resource‐limited settings. The need for a systems approach that considers governance, financing, health information systems, human resources, and access to medicines to provide comprehensive and accessible stroke care has previously been called for by care providers in the Philippines [5].

Given the financial limitations, innovative schemes assessed in other LMICs (such as train‐the‐trainer, digital health technologies and self‐management programmes) could be trialled in the Philippines. This would require collaboration with other LMICs with experience in the relative success of different schemes, and building research capacity within the Philippines to facilitate the contextual adaptation and implementation within the Philippines setting. An understanding of the cost‐effectiveness of stroke rehabilitation would provide the impetus to include this support under the current UHC.

### 4.1 Summary of Recommendations

The recommendations resulting from the survey and interview findings are summarised in Table 7. The overarching need for more accessible and equitable care is central to these recommendations; this can be facilitated by sustained government support, inter‐agency collaboration, community engagement, and the implementation of evidence‐based and cost‐effective community rehabilitation initiatives.

**TABLE 7:** Summary of recommendations based on the study findings.

Stroke‐specific policies and guidelines in line with UHC, that extend beyond acute care and reflect which stroke care services can be provided at the primary care and district/provincial hospital contexts
Adoption and implementation of stroke policies at the local (LGU) level, facilitated by strong leadership and engagement with communities to understand their needs
Improved stroke care workforce, infrastructure, and resource availability—including specialist roles (e.g., neurologists, speech and language therapists), stroke‐ready hospitals, and speciality brain health facilities, focussing on the LGU level
Expansion of PhilHealth stroke and rehabilitation benefit packages, incorporating the full costs of therapy and rehabilitation (including travel costs)
Improved and expanded health information systems and databases to capture accurate national data on stroke care and outcomes (e.g., Philippine Neurological Association One Database‐Stroke)
Initiatives to reduce inequities and improve access to care (e.g., Hub‐and‐Spoke Stroke Care Systems, Community‐Based Inclusive Development programmes)
Holistic, evidence‐based, cost‐effective, sustainable community rehabilitation schemes (e.g., telerehabilitation, train‐the‐trainer, self‐management programmes)
Focus on health promotion, education and preventive care, with emphasis on management of non‐communicable diseases
Multi‐sector collaboration and task forces, including government and non‐government agencies, and other LMICs

### 4.2 Strengths and Limitations

The study had several strengths, including the use of mixed methods to build a more complete picture of the perspectives of government officials and organisational leaders. The widespread sampling and selection resulted in the inclusion of a diverse range of stakeholders from various regions across the Philippines.

A limitation was that different participants completed the survey and undertook the interviews. This was the most practical method as government officials and organisational leaders had limited time to participate in research, however it would be interesting to take a more sequential explanatory approach to data collection to explore the views of the survey respondents in more depth. The number of interviews was small and may be subject to selective bias; it was not possible to interview the leaders of all relevant organisations (only approximately a third of those invited agreed to take part) and therefore generalisability may be limited. However, this is only one component of the wider TULAY project in which the perspectives of multiple stakeholders will be explored, including people with stroke, household carers, and healthcare providers. The findings from surveys and interviews with these additional stakeholders will be reported in separate upcoming publications, with the different stakeholder needs subsequently collated and triangulated to inform the co‐design of a novel community‐based support and training programme for stroke.

## 5 Conclusions

In conclusion, the findings underscore the pressing need for a comprehensive revisiting of stroke care policies in the Philippines, highlighting significant gaps in service delivery and equity. A multi‐sector, community‐focused approach, particularly through initiatives like CBID, is essential for improving access to rehabilitation services, and promoting health literacy among people with stroke and their families. Ultimately, fostering collaboration among stakeholders, and implementing robust, evidence‐based policies will be critical in addressing the challenges faced by stroke care systems, ensuring that all individuals receive equitable and effective support.

## Author Contributions

The authors confirm contribution to the paper as follows: study conception and design: June Ann De Vera, Lorraine Faeldon, Bridie Kent, Angela Logan, Aira Ong, Jonathan Marsden. data collection: June Ann De Vera, Lorraine Faeldon. analysis and interpretation of results: Sarah Buckingham, Aira Ong, Nena Marie Santos. draft manuscript preparation: led by Sarah Buckingham, with all authors contributing to writing, providing critical feedback, reviewing and approving the final version.

## Ethics Statement

The study was reviewed and approved by the Philippines Department of Health Single Joint Research Ethics Board (SJREB, Ref: SJREB‐2023‐85) and the University of Plymouth Faculty of Health Research Ethics and Integrity Committee (Ref: 2024‐4703‐5965). The standard ethical principles for studies involving human participation, including ICH‐GCP and the Declaration of Helsinki were applied.

## Conflicts of Interest

The authors declare no conflicts of interest.

## Supporting information

Supporting Information S1

Supporting Information S2

Supporting Information S3

## Data Availability

The data that support the findings of this study are available from the corresponding author upon reasonable request.
