# Supplementary material for: A Multi‐Sector Mixed Methods Study of Stroke Services in the Philippines: Insights From Government Officials and Organisational Leaders
Source: Int J Health Plann Manage. 2025 Apr 21;40(5):1033–47. doi: 10.1002/hpm.3939 (PMC12411687; doi:10.1002/hpm.3939)
Supplement: Supplementary file 1 — Supporting Information S1 [file HPM-40-1033-s002.docx]

GRAMMS Checklist - O'Cathain A, Murphy E, Nicholl J. The quality of mixed methods studies in health services research. J Health Serv Res Policy. 2008;13(2):92-98

| **Reporting Item** | **Where in Manuscript (page numbers)** |
| --- | --- |
| (1) Describe the justification for using a mixed methods approach to the research question | Page 7 (Overview of methods) and page 10 (Data integration) |
| (2) Describe the design in terms of the purpose, priority and sequence of methods | Page 7 (Overview of methods) |
| (3) Describe each method in terms of sampling, data collection and analysis | Survey methods page 7-9  Interview methods page 9-10 |
| (4) Describe where integration has occurred, how  it has occurred and who has participated in it | Page 10 (Data integration) |
| (5) Describe any limitation of one method associated with the presence of the other method | Page 26-27 (Strengths and limitations) |
| (6) Describe any insights gained from mixing or integrating methods | Page 23-26 (Discussion)  Also described throughout the Results section (page 10-23) |
