# Supplementary material for: A Multi‐Sector Mixed Methods Study of Stroke Services in the Philippines: Insights From Government Officials and Organisational Leaders
Source: Int J Health Plann Manage. 2025 Apr 21;40(5):1033–47. doi: 10.1002/hpm.3939 (PMC12411687; doi:10.1002/hpm.3939)
Supplement: Supplementary file 2 — Supporting Information S2 [file HPM-40-1033-s003.docx]

**Paper survey for Local Officials & Government Employees**

**(Provincial, Municipal and barangay level)**

**Title of Study:** Understanding stroke care and rehabilitation in the Philippines: A multi-sector survey and interview approach

**What is this survey for?**

The aim of this survey is to capture the current support available for rehabilitation in the Philippines, assessing from barangay to national level the structure of stroke care with a focus on rehabilitation.

**Will my involvement in the survey be kept confidential?**

Yes. The survey is anonymous, and all responses are treated as strictly confidential. No results will be used that could identify or be linked to individuals and there will be no consequences to you as an individual for your participation.

All questions in this survey are voluntary, so you may leave questions blank that you do not wish to answer, and you can discontinue the survey at any point. As the survey is anonymous, we cannot withdraw your submission once completed as we will not be able to identify your response.

If you have any questions or concerns about the survey, please contact the survey administrators at ibeht.tulayproject@dlsu.edu.ph

**Consent**

If you agree to take part in the survey, please tick all the boxes below:

| I agree to take part in this survey. | 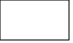 |
| --- | --- |
| I have read and understood the information sheet (dated 03/05/23 Version 1) and I have had the opportunity to ask questions about the study | 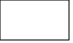 |
| I understand that the survey is entirely voluntary and that my response is anonymous | 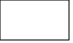 |

Section 1: About your role (Local Officials & Government Employees)

- 1. *What is your current role?*

☐ *DoH official*

☐ *Local Chief Executive (Mayor, Governor)*

☐ *Local policymaker or councilor*

☐ *LGU employee (MHO, PSWDO, PDAO, etc)*

☐ *National Council for Disability Affairs (Under the Department of Social Welfare)*

☐ *Non-Government Agencies*

☐ *Other* (please state):___________

- 1. *Where do you work?* Please state:___________________________
  2. *Which branch do you work in?*

☐ *Legislative*

☐ *Executive*

☐ *Both*

☐ *Other* (please state):___________

- 1. *What level of government do you work at?*

☐ *National*

☐ *Regional*

☐ *Provincial*

☐ *Municipal/City*

☐ *Barangay*

☐ *Other (please state):___________*

- 1. *How long have you worked in your current position (in years)? ___________*
  2. *Are you currently involved in determining the budget for healthcare spending?*

☐*Yes* ☐*No*

- 1. *What areas of improvement most matter to you with a community-based rehabilitation programme? (order from 1-4, 1 being the most important) [*ASK RESPONDENT TO RANK 1- MOST IMPORTANT 4- LEAST IMPORTANT]

*____ Improvement in people’s quality of life and happiness*

*____ Improvement in ability to do things*

*____ Ability to return to work*

*____ Decrease in burden for carers and family members*

- 1. *What information is useful to you before adopting a healthcare policy? (order from 1-4, 1 being the most useful) [*ASK RESPONDENT TO RANK 1- MOST IMPORTANT 4- LEAST IMPORTANT]

*____ Knowledge of the impact of the condition on a person’s function and quality of life?*

*____ Knowledge of the impact of the condition on a person’s family in terms of ability to continue working?*

*____ Knowledge of how many people have the condition in your area?*

*____ Knowledge of potential healthcare resources and costs?*

*Other information that is useful for adopting a healthcare policy (please state):*

*______________________________________________________________*

- 1. *Where would you rank your current interest in community-based rehabilitation projects? On a scale of 0 (not interested) to 10 (very interested) (circle one)*

| 0 | 1 | 2 | 3 | 4 | 5 | 6 | 7 | 8 | 9 | 10 |
| --- | --- | --- | --- | --- | --- | --- | --- | --- | --- | --- |

- 1. *Could you rank these areas in terms of your perceived local importance? (order from 1-7, 1 being the most important) [*ASK RESPONDENT TO RANK 1- MOST IMPORTANT 7- LEAST IMPORTANT]

*____ Infrastructure projects*

*____ Economic development*

*____ Disaster mitigation, preparedness and response*

*____ Peace and order/security*

*____ Food security*

*____ Healthcare prevention*

*____ Healthcare rehabilitation after disease or injury*

- 1. *What factors would you consider before adopting a new health policy? (tick all that apply) [MULTIPLE RESPONSES AND PROMPTED]*

☐ *Government Policy*

☐ *Evidence of clinical effectiveness / importance*

☐ *Community need*

☐ *Budget availability / allocation*

☐ *Space and facilities*

☐ *Availability of trained healthcare workers*

☐ *Other (please state): _________________________*

- 1. *What factors influence you adopting a new ordinance? (order from 1-3, 1 being the most important) [*ASK RESPONDENT TO RANK 1- MOST IMPORTANT 3- LEAST IMPORTANT]

*_____Local people’s voice*

*_____Policy mentioned in your local platform*

*_____Government policy*

**Final section**

We are interested in gathering more in-depth information about people’s stroke experiences. If you would be interested in assisting us with future work packages involving the implementation of our proposed programs and activities, then please add your contact details below. These activities would occur in 2024. We will keep your contact details secure and not pass them on to any third party.

Please fill in as many sections as possible.

| Name |  |
| --- | --- |
| Address |  |
| E mail |  |
| Phone |  |

Preferred time for contact/interview (tick all that apply):

☐ Morning ☐ Afternoon ☐ Evening

Preferred day(s) for contact/ interview (tick all that apply):

☐ Monday ☐ Tuesday ☐ Wednesday ☐ Thursday ☐ Friday

**Thank you for completing the survey!**
