# Supplementary material for: A Multi‐Sector Mixed Methods Study of Stroke Services in the Philippines: Insights From Government Officials and Organisational Leaders
Source: Int J Health Plann Manage. 2025 Apr 21;40(5):1033–47. doi: 10.1002/hpm.3939 (PMC12411687; doi:10.1002/hpm.3939)
Supplement: Supplementary file 3 — Supporting Information S3 [file HPM-40-1033-s001.docx]

Interview topic guide: Policy Makers WP2 (English version)

Structure of care (Macro level)

• Semi-structured interview

• DOH representatives, PhilHealth, PARM, PPTA, associations/ organizations Stroke Society, charity, NGOs, churches (national level)

**PLEASE MAKE SURE THEY HAVE SIGNED THE INFORMED CONSENT**

**JISC 2.0 ICF FOR INTERVIEW:**

https://plymouth.onlinesurveys.ac.uk/wp2-interview-consent-form https://plymouth.onlinesurveys.ac.uk/tulay-wp2-interview-consent-form-tagalog

Researcher introduction:

- Introduce self and TULAY Project [Sub-lead as main interviewer & RA as note-taker]
- Briefly explain the purpose of the interview and what topics will be covered
- Check how much time the participant has available
- Check they have read and understood the study information – do they have any questions?
- State the interview is voluntary and they can withdraw at any time or request removal of data before it is analysed
- Highlight data protection – data (contact details, interview recording) will be stored securely; personal details will not be shared outside the research team; any names/local places/hospitals you mention will be removed from interview transcripts and reports
- Check consent form has been completed – if not, do this now
- Re-confirm consent for video- or audio-recording the interview (log interview start and end time) and start recording

***Note for researchers:*** Focus on the questions (middle column) and use the prompts as necessary.

| **Topic** | **Questions** | **Prompts** |
| --- | --- | --- |
| **Demographics and Roles** | | |
| About you and your roles – Demographics, Sex, Age | 1. Basic background information 2. “Could you describe your job role?” 3. What area of the Philippines do you cover – is this at a national, provincial or regional level? | *How long have you had this job / how long have you been in your present position?*  *For regional - how long have you been serving this community? ** |
| **Structure and Organisation of Stroke Care** | | |
| “This section will ask about your opinion of the current structure and organisation of stroke care” | 4. Can you describe how you perceive stroke related services are currently organised in the Philippines?  a. Could you tell me how a person with an acute stroke who requires an emergency appointment is managed?  b. Could you describe the current state of rehabilitation services for people with  stroke?  c. Could you describe social care for people with stroke?  i. Who undertakes this usually? | *Do you feel there are any regional variations in stroke care?* |
|  | 1. What are the main policy documents governing the running of stroke services at a regional level? * 2. Are there other existing policies that govern stroke care? |  |
|  | 1. How do government-run and private organisations currently co-ordinate care for people with a stroke? |  |
|  | 1. Do you know of any future plans at national or regional level that could affect the delivery of stroke care? |  |
|  | 1. For children who have had a stroke is there any difference in the pattern of stroke care delivery across the country? |  |
| **Perceived changes to stroke care, barriers and facilitators** | | |
| “This section will ask about your perception of whether and how stroke services should change. It  will break stroke care into acute care, rehabilitation and social care in the community”. | 10. Do you know of any examples of innovation or best practice for stroke care within the Philippines?  a. What makes this an example of best practice?  b. Do you feel that this could be applied to other areas of the Philippines? |  |
|  | 11. What do you feel are currently the main issues in **acute stroke care**?  a. Do you have any ideas about how acute stroke care can be improved in the short  and long term?  b. What do you feel are the main barriers towards improving acute stroke care?  c. Are there any factors that can facilitate a change in stroke care?  *Note; be sure to capture both the long term and short term answers, follow up |  |
|  | 12. What do you feel are the main issues currently in the **rehabilitation** of a person with a stroke?  a. Do you have any ideas about how stroke rehabilitation can be improved in the short and long term?  b. What do you feel are the main barriers towards improving stroke rehabilitation?  c. Are there any factors that can facilitate a change in stroke rehabilitation? |  |
|  | 13. What do you feel are the main issues currently in the **social care** of a person with a stroke in the community?  a. Do you have any ideas about how social care can be improved in the short  and long term?  b. What do you feel are the main barriers towards improving social care?  c. Are there any factors that can facilitate a change in social care? | *Social care: would you know of any community programs being employed for stroke care?* |
| **Financial Aspects of Stroke Care** | | |
|  | 14. For Acute:   1. What are the existing financial policies that are currently implemented for acute stroke care? 2. What are the existing financial programs that are currently implemented for acute stroke care? 3. What are the future plans for financial policies and programs in acute stroke care? |  |
|  | 15. For Rehabilitation:   1. What are the existing financial policies that are currently implemented for rehabilitation? 2. What are the existing financial programs that are currently implemented for rehabilitation? 3. What are the future plans for financial policies and programs in rehabilitation? |  |
| Closing: Do you have any other issues and concerns that you want to be part of the plan or implemented for stroke care within the context of the universal health care law? | | |

Researcher summary:

- Summarise key points discussed during the interview.
- Ask if there is anything they would like to discuss further and state that you are available to discuss the study, especially if there are any issues that arose or discussion points that may have caused distress.
- Check they are still happy for the recorded interview to be used for the purposes stated on the consent form.
- Thank the participant for their time and insights.
- Ask if they would like to receive a summary of study findings.
